# High-sensitivity assay for monitoring *ESR1* mutations in circulating cell-free DNA of breast cancer patients receiving endocrine therapy

**DOI:** 10.1038/s41598-018-22312-x

**Published:** 2018-03-12

**Authors:** Laura Lupini, Anna Moretti, Cristian Bassi, Alessio Schirone, Massimo Pedriali, Patrizia Querzoli, Roberta Roncarati, Antonio Frassoldati, Massimo Negrini

**Affiliations:** 1 Università di Ferrara, Dipartimento di Morfologia, Chirurgia e Medicina Sperimentale, Via Luigi Borsari, 46, 44121 Ferrara (FE), Italy; 2 Azienda Ospedaliero Universitaria di Ferrara, Divisione di Oncologia clinica, via Aldo Moro, 8, 44124 Cona (FE), Italy; 3 Azienda Ospedaliero Universitaria di Ferrara, Unità di Anatomia Patologica, via Aldo Moro, 8, 44124 Cona (FE), Italy; 4 Institute of Genetics and Biomedical Research, Consiglio Nazionale delle Ricerche, Milano (MI), Italy

## Abstract

Approximately 70% of breast cancers (BCs) express estrogen receptor alpha (ERα) and are treated with endocrine therapy. However, the effectiveness of this therapy is limited by innate or acquired resistance in approximately one-third of patients. Activating mutations in the *ESR1* gene that encodes ERα promote critical resistance mechanisms. Here, we developed a high sensitivity approach based on enhanced-ice-COLD-PCR for detecting *ESR1* mutations. The method produced an enrichment up to 100-fold and allowed the unambiguous detection of *ESR1* mutations even when they consisted of only 0.01% of the total *ESR1* allelic fraction. After COLD-PCR enrichment, methods based on next-generation sequencing or droplet-digital PCR were employed to detect and quantify *ESR1* mutations. We applied the method to detect *ESR1* mutations in circulating free DNA from the plasma of 56 patients with metastatic ER-positive BC. Fifteen of these patients were found to have *ESR1* mutations at codons 536–538. This study demonstrates the utility of the enhanced-ice-COLD-PCR approach for simplifying and improving the detection of *ESR1* tumor mutations in liquid biopsies. Because of its high sensitivity, the approach may potentially be applicable to patients with non-metastatic disease.

## Introduction

Breast cancer (BC) is the most commonly diagnosed neoplastic disease in women worldwide and has a high incidence in Western countries where it is the second leading cause of cancer-related death^1^.

Approximately 70% of breast tumors express estrogen receptor alpha (ERα) at diagnosis; proliferation and survival of neoplastic cells are dependent on estrogen stimulation^2^. Patients with these cancers are administered endocrine-based therapies that stop or slow tumor growth via various mechanisms of action. Therapeutic agents include tamoxifen, a specific estrogen antagonist; aromatase inhibitors (AIs), which suppress estrogen production; and fulvestrant, which promotes ERα degradation.

Antiestrogenic drugs produce survival benefits in patients with BC; however, one-third of patients develop resistance to therapy. Missense mutations in the *ESR1* gene, which encodes ERα, represent an important mechanism leading to endocrine resistance^3^. Most mutations of the *ESR1* gene are found in codons 536–538. These mutations have been shown to promote ERα transcriptional activity in an estrogen-independent manner^4^. Among the mutations, Y537S, Y537N, Y537C, and D538G represent more than 80% of the abnormalities found in resistant cases^5,6^.

Such mutations have been identified in approximately 15–20% of ER-positive (ER+) metastatic lesions from patients treated with endocrine therapy, but rarely in primary tumors. It is therefore believed that these alterations are selected from rare mutant clones to confer resistance to therapy and possibly favor the development of metastatic disease^6^. It is thus essential to detect these mutations as soon as possible to select the best therapeutic options.

Tissue biopsy is generally not a suitable approach for the frequent monitoring of disease because the invasive nature of the required procedures; moreover, mutation could be missed because of tumor heterogeneity. These limitations can be overcome by a liquid biopsy approach, based on analysis of circulating cell-free DNA (cfDNA) to monitor patients with advanced cancer during clinical follow-up. Such patients have cfDNA that is often enriched with tumor DNA (ctDNA), which makes it possible to pinpoint genetic or epigenetic changes that are present in tumor cells^7–9^. Assessing ctDNA is minimally invasive and, more importantly, can detect mutations from hidden metastases and genetically heterogeneous tumors.

The technical challenges of this type of analysis are related to the low amount of cfDNA present in plasma as well as the low proportion of ctDNA. Therefore, high sensitivity of detection is essential. Several studies have been published in recent years using next-generation sequencing (NGS), real-time PCR, or droplet digital PCR (ddPCR) to perform liquid biopsy tests aimed at revealing specific cancer-associated changes in cfDNA^10–14^. Some of these studies have been aimed at identifying *ESR1* mutations in the cfDNA of patients with endocrine-resistant breast cancer^4,15–19^.

To improve the sensitivity of mutation detection, methods have been developed to enrich low-frequency allelic variants (COLD-PCR and its derivatives)^20–22^. In particular, such approaches have been reported to enrich the *BRAF* and *KRAS* variants associated with cancer^21,23^. In this study, we developed an enhanced-ice-COLD-PCR (E-ice-COLD-PCR)-based method for the enrichment of *ESR1* gene mutations at codons 536–538. We demonstrated that the use of this approach consistently improved detection of *ESR1* alterations compared to other currently employed methods. We tested this approach in a large group of patients with metastatic BC to investigate its potential clinical applicability.

## Results

### *ESR1* gene mutations in primary and metastatic breast cancer lesions

With the aim of establishing a sensitive method for the detection of *ESR1* mutations in cfDNA, we identified *ESR1* mutant alleles by investigating tumor tissue samples from a cohort of 40 patients with metastatic BC (Table 1). All patients were diagnosed with ER+ BC and treated with endocrine therapy. None of the patients had metastasis at diagnosis. Primary tumor samples (N = 40) and metastatic lesions (N = 47) were from matched patients. In these samples, we examined mutations to codons 536–538 of the *ESR1* gene using Sanger sequencing. We identified *ESR1* mutations in 6 metastases (none of which were in primary tumors): Y537S was found in 3 samples, D538G in 2 samples, and Y537C in 1 (Table 1).

**Table 1:** ESR1 variations in codons 536–538 in primary tumors and metastasis of 40 BrCa patients.

Patient	Tumor tissue	Metastasis site	Date	ER	PR	HER2	MIB1	ESR1 status (codons 536–538)
S-26	primary		23/01/2013	93%	72%	1+	42%	WT
	metastasis	liver	17/03/2015	95%	10%	1+	20%	WT
S-27	primary		19/04/2001	86%	84%	0	7%	WT
	metastasis	skin	16/09/2008	94%	56%	0	24%	WT
S-28	primary		23/12/2006	100%	90%	1+	20%	WT
	metastasis	liver	28/10/2013	99%	30%	1+	35%	WT
S-30	primary		16/01/2014	56%	35%	1+	45%	WT
	metastasis	skin	31/03/2015	95%	41%	2+	48%	WT
S-31	primary		17/05/2013	96%	82%	1+	15%	WT
	metastasis	skin	14/09/2015	99%	98%	2+	24%	Y537S
S-32	primary		31/05/2011	64%	41%	0	22%	WT
	metastasis	skin	04/03/2015	85%	0%	0	2%	WT
S-33	primary		12/05/2010	22%	0%	1+	84%	WT
	metastasis	skin	11/05/2011	25%	0%	NA	NA	WT
S-34	primary		11/02/2008	60%	10%	3+	50%	WT
	metastasis	skin	05/05/2012	4%	0%	3+	57%	WT
S-35	primary		05/08/2009	96%	8%	1+	50%	WT
	metastasis	brain	31/07/2014	94%	0%	1+	27%	WT
S-36	primary		07/10/2010	75%	23%	1+	2%	WT
	metastasis 1	ovary	31/05/2013	90%	71%	1+	19%	WT
	metastasis 2	ovary	31/05/2013	NA	NA	NA	NA	WT
S-37	primary		16/10/2007	100%	100%	0	1%	WT
	metastasis	ovary	05/10/2011	95%	pos	NA	NA	WT
S-38	primary		04/04/2013	78%	2%	1+	39%	WT
	metastasis	liver	14/09/2015	30%	2%	1+	43%	WT
S-39	primary		04/04/2006	100%	76%	3+	17%	WT
	metastasis 1	liver	18/04/2013	98%	0%	3+	15%	WT
	metastasis 2	liver	24/02/2014	99%	0%	3+	20%	D538G
S-40	primary		15/06/2006	99%	80%	0	26%	WT
	metastasis	liver	10/12/2014	0%	25%	0	40%	WT
S-41	primary		25/02/2009	48%	46%	3+	42%	WT
	metastasis	liver	07/07/2014	91%	79%	3+	48%	WT
S-42	primary		03/04/2012	53%	4%	3+	19%	WT
	metastasis	liver	26/03/2014	58%	0%	3+	30%	WT
S-43	primary		13/11/2009	88%	26%	2+	16%	WT
	metastasis	liver	26/11/2012	12%	0%	1+	40%	WT
S-44	primary		23/02/2009	45%	48%	1+	25%	WT
	metastasis	liver	13/05/2011	74%	69%	1+	65%	WT
S-45	primary		10/03/2009	98%	56%	1+	12%	WT
	metastasis	liver	26/04/2011	pos	NA	NA	NA	WT
S-46	primary		18/12/2006	99%	0%	3+	25%	WT
	metastasis	brain	26/02/2011	42%	0%	3+	38%	WT
S-47	primary		30/05/2002	99%	40%	0	5%	WT
	metastasis	ovary	20/09/2013	99%	99%	1+	10%	Y537S
S-51	primary		28/03/2007	99%	99%	0	5%	WT
	metastasis	lung	27/12/2013	98%	95%	0	18%	Y537C
S-52	primary		13/03/2008	89%	0%	1+	16%	WT
	metastasis	liver	20/04/2009	15%	NA	NA	NA	WT
S-53	primary		25/03/2005	NA	NA	NA	NA	WT
	metastasis 1	ovary	20/04/2011	55%	21%	0	5%	WT
	metastasis 2	liver	31/10/2012	98%	15%	0	60%	Y537S
S-54	primary		30/12/2010	31%	30%	3+	45%	WT
	metastasis 1	brain	04/10/2012	0%	pos	pos	NA	WT
	metastasis 2	brain	03/09/2014	5%	3%	3+	30%	WT
	metastasis 3	brain	16/04/2015	1%	0%	3+	35%	WT
S-55	primary		25/05/2006	99%	63%	0	41%	WT
	metastasis	skin	04/09/2007	90%	45%	NA	NA	WT
S-56	primary		22/04/2008	99%	99%	0	2%	WT
	metastasis	ovary	30/11/2013	pos	pos	0	NA	WT
S-57	primary		04/04/2001	87%	93%	0	40%	WT
	metastasis	lung	19/01/2015	98%	35%	1+	60%	WT
S-58	primary		16/05/2012	75%	46%	3+	33%	WT
	metastasis	brain	12/11/2014	pos	NA	NA	NA	WT
S-59	primary		19/12/2008	55%	36%	1+	27%	WT
	metastasis	liver	16/04/2010	52%	27%	1+	14%	WT
S-60	primary		12/12/2006	98%	0%	2+	45%	WT
	metastasis	liver	17/11/2015	98%	0%	2+	45%	WT
S-61	primary		06/09/2006	99%	91%	0	26%	WT
	metastasis	liver	21/03/2014	99%	30%	1+	35%	WT
S-62	primary		20/11/2001	NA	NA	NA	NA	WT
	metastasis	ovary	06/07/2010	pos	pos	NA	NA	WT
S-63	primary		13/06/2001	98%	96%	0	17%	WT
	metastasis	lung	23/11/2009	75%	NA	1+	NA	WT
S-64	primary		20/02/2013	1%	0%	1+	51%	WT
	metastasis	lung	22/04/2015	5%	neg	1+	NA	WT
S-65	primary		05/10/2011	26%	0%	3+	35%	WT
	metastasis	liver	28/03/2014	21%	0%	3+	47%	WT
S-66	primary		21/07/2004	53%	46%	NA	61%	WT
	metastasis	lung	28/12/2009	pos	pos	0	NA	WT
S-67	primary		29/05/2000	96%	66%	0	61%	WT
	metastasis	lung	06/08/2010	pos	NA	NA	NA	WT
S-68	primary		19/04/2010	96%	87%	1+	33%	WT
	metastasis	skin	31/05/2012	pos	pos	NA	NA	WT
S-69	primary		04/05/2010	98%	12%	0	42%	WT
	metastasis	liver	03/06/2014	pos	NA	0	NA	D538G

pos: > 10% of positive tumor cells.

NA: not available.

**Figure 1:**
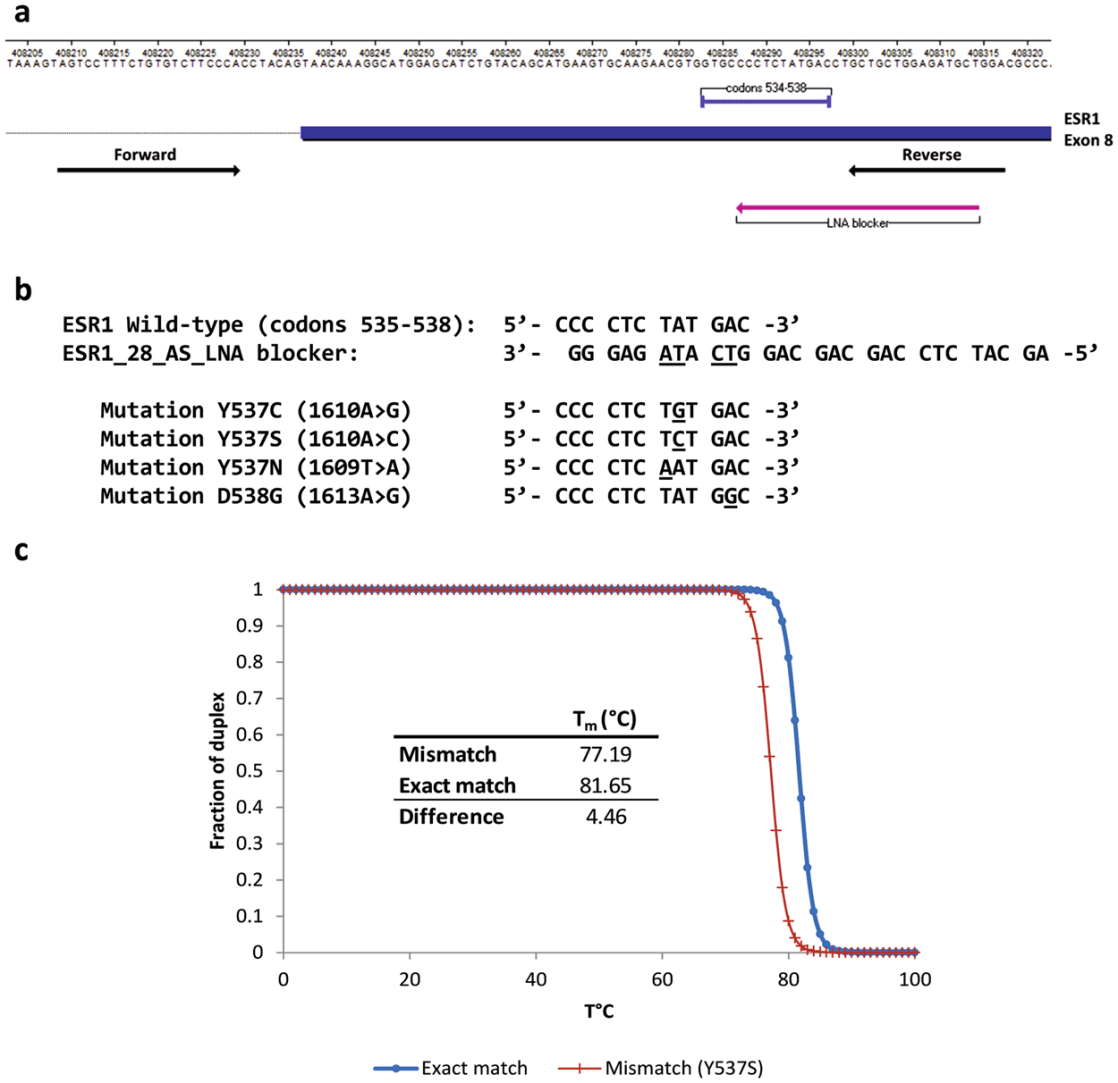
*ESR1* enhanced-ice-COLD PCR assay design. (**a**) Location of *ESR1*_109F (forward) and *ESR1_*109R (reverse) primers, as well as the *ESR1*_28_AS_LNA blocker in the *ESR1* gene (exon 8). (**b)** Nucleotide sequence of the *ESR1*_28_AS_LNA blocker. Locked nucleic acid (LNA) nucleotides (underlined letters) correspond to the most frequently mutated nucleotides in the Y537 and D538 codons (underlined letters). (**c)** Theoretical melting curves and melting temperatures (Tm) of the LNA-blocker/wildtype *ESR1* and LNA-blocker/Y537S *ESR1* duplex.

DNA samples with *ESR1* mutations were employed to develop a method for the specific enrichment of mutant alleles present in the *ESR1* 536–538 codons. Based on the E-ice-COLD-PCR method^21,23,24^, we designed primers for PCR amplification as well as a partially overlapping oligonucleotide blocker (Fig. 1a,b). The blocker was designed to include Locked nucleic acid (LNA) modified-nucleotides at the putative mutant codons and a phosphate group at the 3′-end to block its extension. The melting temperature of the blocker was 81.7 °C if matched to a wildtype sequence, but lower (77.2 °C for Y537S) if a mutation was present (Fig. 1c). The different melting temperatures functioned to block or limit the amplification of the wildtype sequence and thereby favor the enrichment of any present mutant allele.

To test the ability of the method to enrich mutant alleles, DNA from mutant samples (Y537S, Y537C, and D538G) was diluted in normal DNA to achieve allelic frequencies of 1% and 0.5%. After performing E-ice-COLD-PCR, amplicons were analyzed by NGS to measure the achieved frequency of mutation. All 3 mutations were found to be considerably enriched (9–70-fold). No mutated *ESR1* was amplified in SW480 colorectal cancer cell DNA, which was used as a negative control (Table 2). The concentration of the blocker that produced the highest Y537S mutation enrichment was 80 nM (Supplementary Figure 1).

**Table 2:** Enrichment of ESR1 hotspot mutations after E-Ice-COLD PCR.

Sample	ESR1 status	Before E-Ice-COLD	After E-Ice-COLD	Fold enrichment	Sequencing depth
		Variant frequency %	Variant frequency %		
S-31	Y537S	1.0	44.2	**44**.**2**	1060
S-31	Y537S	0.5	35.4	**70**.**8**	1959
S-51	Y537C	1.0	7.6	**7**.**6**	396
S-51	Y537C	0.5	13.3	**26**.**6**	2033
S-39	D538G	1.0	9.5	**9**.**5**	1006
S-39	D538G	0.5	6.6	**13**.**2**	665
SW480	WT	0.0	0.0	**0**.**0**	1603

**Figure 2:**
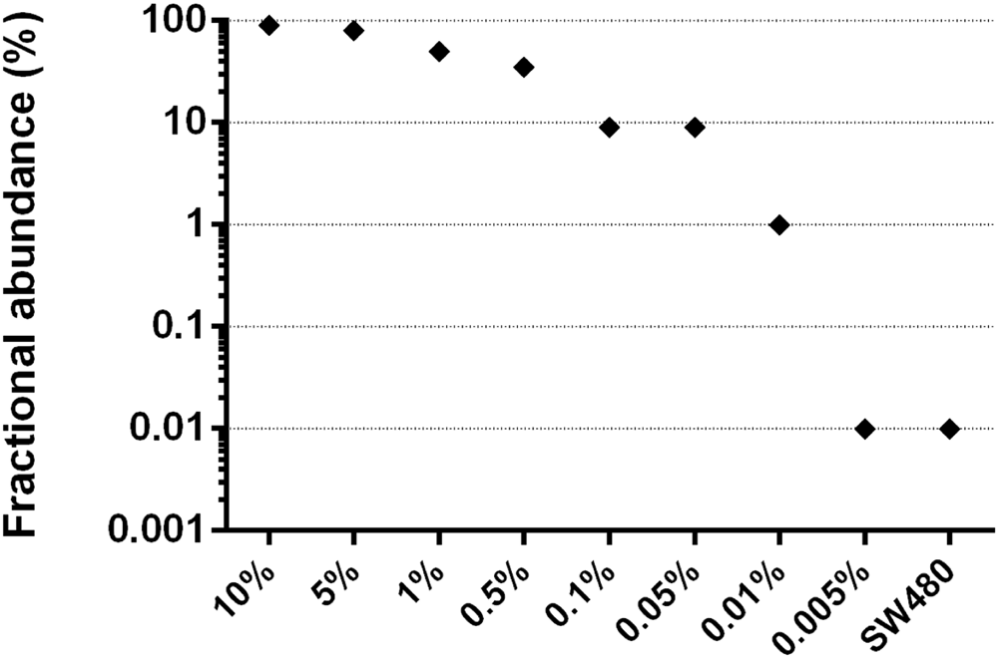
Sensitivity of *ESR1* enhanced-ice-COLD-PCR assay. Y537S alteration was diluted in wild type DNA to attain mutation fractions ranging from 10% to 0.005%. All dilutions and SW480 cell wild type DNA were subjected to enhanced-ice-COLD-PCR followed by droplet digital PCR analysis. The detected Y537S fractional abundance was enriched 100-fold compared to the initial abundance in templates, allow the detection of the Y537S mutation at a 0.01% dilution (i.e., 1 mutant in 10,000 molecules).

After demonstrating the potential of the method to increase the frequency of *ESR1* mutant alleles, we next evaluated its lower limit of detection by designing fluorescent probes capable of discriminating the Y537S mutant from wild type DNA. We serially diluted the Y537S mutant DNA in normal DNA; the smallest dilution was 0.005% (1 mutant among 20,000 molecules). All dilutions were subjected to E-ice-COLD-PCR in duplicate, and the resultant amplicons were quantified by droplet-digital PCR (ddPCR) using fluorescence-specific probes for either the Y537S or wild type allele. The mutant allele was detected at a minimum original dilution of 0.01% (i.e., detected at 1%, with 100-fold enrichment) after the application of E-ice-COLD-PCR (Fig. 2).

### *ESR1* gene mutation in plasma of breast cancer patients

To test the assay in a clinical setting, we analyzed DNA from the plasma of 56 patients with metastatic ER+ breast cancer. We performed E-ice-COLD amplification in the hotspot region of the *ESR1* gene. The resulting amplicons were analyzed using both ddPCR (for the Y537S variant) and NGS for all mutation types. Overall, 15 patients (27%) were found to have a mutation in codons 536–538 (Table 3 and Supplementary Figure 2). The results for the detection of the Y537S variant obtained with both methods were consistent (Table 3). Additionally, the experiment also proved that specificity of the method of detection based on ddPCR labeled-probe was 100%, since not only negative samples remained negative but also mutants other than Y537S ESR1 were negative when assayed for the Y537S mutation.

**Table 3:** ESR1 variants in plasma cfDNA of metastatic BC patients.

Sample	Blood sampling date	Mutation in plasma after E-Ice-COLD					Metastasis biopsy date	Mutation in Metastasis
		Variant^*a*^		Sequencing depth	NGS (Freq%)	ddPCR Y537S (Freq%)		
S-26	19-Jun-15	None		2695	—	—	17-Mar-15	None
S-26	13-May-16	p.Y537S	c.1610A > C	1995	94	93.8		
S-26	28-Mar-17	p.Y537S	c.1610A > C	1994	83	80		
S-27	17-May-16	None		1984	—	—	16-Sep-08	None
S-28	17-May-16	p.L536H	c.1607T > A	1881	34	—	28-Oct-13	None
S-29	27-May-16	None		1970	—	—		—
S-31	15-Sep-16	p.Y537S	c.1610A > C	1534	23	25	14-Sep-15	Y537S
S-51	1-Sep-16	None		1988	—	—	27-Dec-13	Y537C
S-60	29-Aug-16	None		525	—	—	17-Nov-15	None
S-74	20-Sep-16	None		1993	—	—		—
S-80	2-Nov-16	None		1936	—	—		—
S-81	2-Nov-16	p.Y537S	c.1610A > C	1443	81	84		—
S-84	15-Nov-16	None		1991	—	—		—
S-85	15-Nov-16	None		1476	—	—		—
S-86	15-Nov-16	p.Y537S	c.1610A > C	1988	16	16		—
S-87	15-Nov-16	p.Y537S	c.1610A > C	1232	87	88		—
S-88	16-Nov-16	p.Y537S	c.1610A > C	200	18	34		—
S-89	16-Nov-16	None		1987	—	—		—
S-90	16-Nov-16	None		1300	—	—		—
S-91	16-Nov-16	p.D538G	c.1613A > G	1988	12	—		—
S-94	30-Nov-16	p.L536H	c.1607T > A	201	18	—		—
S-96	12-Dec-16	None		1888	—	—		—
S-97	21-Dec-16	None		1997	—	—		—
S-98	16-Dec-16	p.D538G	c.1613A > G	1984	29	—		—
S-99	16-Dec-16	None		1996	—	—		—
S-100	3-Mar-17	None		113	—	—		—
S-101	7-Mar-17	p.D538G	c.1613A > G	1977	12	—		—
S-102	7-Mar-17	p.D538G	c.1613A > G	1992	35	—		—
S-103	16-Mar-17	None		1982	—	—		—
S-104	16-Mar-17	p.Y537H	c.1609T > C	1459	17	—		—
S-105	24-Mar-17	None		1142	—	—		—
S-106	24-Mar-17	None		1995	—	—		—
S-107	24-Mar-17	None		1991	—	—		—
S-108	24-Mar-17	None		1995	—	—		—
S-109	24-Mar-17	None		376	—	—		—
S-110	28-Mar-17	None		135	—	—		—
S-111	28-Mar-17	None		252	—	—		—
S-112	29-Mar-17	p.Y537S	c.1610A > C	1734	40	39		—
S-113	29-Mar-17	None		1931	—	—		—
S-114	29-Mar-17	None		1427	—	—		—
S-115	29-Mar-17	None		851	—	—		—
S-116	4-Apr-17	None		990	—	—		—
S-117	4-Apr-17	p.L536Q	c.1607_1608TC > AG	1889	65	—		—
S-118	5-Apr-17	None		1976	—	—		—
S-119	5-Apr-17	None		495	—	—		—
S-120	6-Apr-17	None		1921	—	—		—
S-121	6-Apr-17	None		1857	—	—		—
S-122	6-Apr-17	None		1811	—	—		—
S-123	7-Apr-17	None		1990	—	—		—
S-124	11-Apr-17	None		1983	—	—		—
S-125	11-Apr-17	None		1999	—	—		—
S-126	11-Apr-17	None		1992	—	—		—
S-127	11-Apr-17	None		1822	—	—		—
S-128	4-May-17	None		1960	—	—		—
S-129	9-May-17	None		1930	—	—		—
S-130	29-May-17	None		987	—	—		—
S-131	29-May-17	None		1561	—	—		—
S-132	29-May-17	None		1362	—	—		—

^*a*^Reference genome: NC_000006.11 (GRCh37/hg19); *ESR1* transcript: NM_000125.3.

In 6 patients (S-26, S-27, S-28, S-31, S-51, and S-60), tumor and metastasis tissues were available among samples initially analyzed for the mutational status of *ESR1*. Four of these patients (S-26, S-27, S-28, and S-60) did not show any *ESR1* mutation, while the remaining 2 patients (S-31 and S-51) showed mutations in samples derived from metastases. Analysis of the corresponding cfDNA revealed what follows: in 3 cases, the results were consistent in that 2 patients (S-27 and S-60) had no mutations while 1 (S-31) had the same mutation in both the metastasis and cfDNA (Y537S). In the remaining 3 cases, 2 patients (S-26 and S-28) exhibited *ESR1* mutations in their cfDNA but not in the original metastasis biopsy, while the remaining patient (S-51) exhibited the opposite situation.

**Figure 3:**
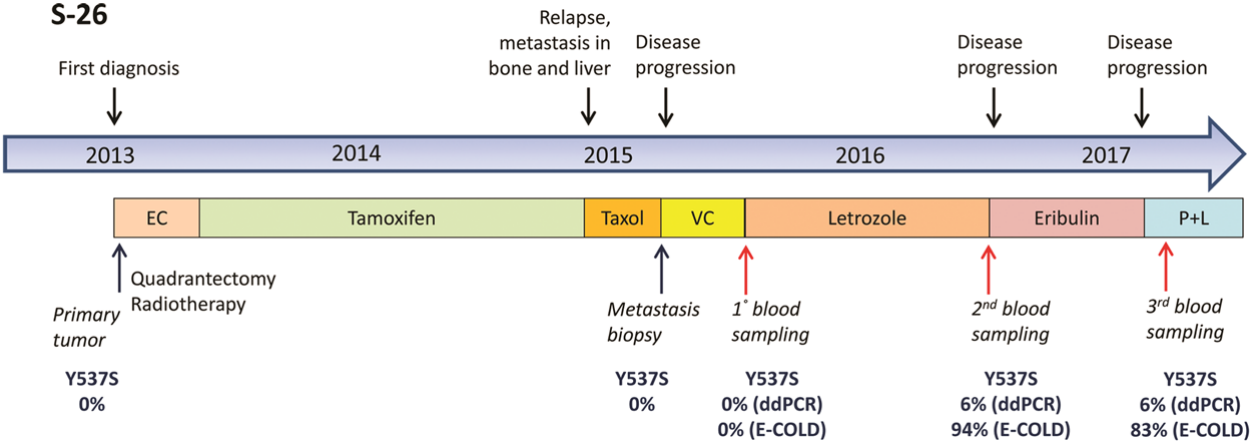
Clinical timeline for patient S-26. The timeline extends from January 2013 (first diagnosis) to March 2017 (last recorded checkup). Lines of treatment, tissue and liquid biopsies, and corresponding results of *ESR1* Y537S mutation analyses (after a regular droplet digital PCR [ddPCR] or after enhanced-ice-COLD-PCR [E-COLD]) are displayed. The percentages of mutations in tissues (primary and metastasis) were assessed by next-generation sequencing and ddPCR analyses. **EC**: Epirubicin/Cyclophosphamide; **VC**: Vinorelbine/Capecitabine; **P + L**: Palbociclib + Letrozole

Patient S-26 had a markedly long interval between biopsy and blood withdrawal. In this patient, multiple blood samplings made it possible to monitor the evolution of the cancer’s status over time. Analysis of metastatic tissue and liquid biopsy samples collected in the spring of 2015 showed that both samples were negative for *ESR1* mutations. One year later (May 2016), the liquid biopsy was positive for the *ESR1* Y537S mutation. The patient was administered letrozole therapy between Spring 2015 and Spring 2016, raising the possibility that this therapy was responsible for selecting the mutant neoplastic clone (Fig. 3).

For patient S-28, a similar situation can be envisioned, since 33 months had elapsed between the liver metastasis biopsy (October 2013) and blood withdrawal (May 2016). Considering that the patient underwent several consecutive lines of endocrine therapy, it is plausible that the *ESR1* mutation was not detectable in October 2013 but was subsequently selected.

Patient S-51 showed an opposite pattern: the liquid biopsy obtained in September 2016 was mutation-negative, while the metastasis as evaluated in December 2013 was positive for the Y537C mutation. The patient was administered AIs until December 2013, and was on fulvestrant therapy starting from January 2014, suggesting that the latter was effective in eliminating cells with *ESR1* mutations.

## Discussion

In recent years, liquid biopsy technology has evolved rapidly because of its great potential and minimal invasiveness. cfDNA can be used to monitor the evolution of mutations associated with neoplastic disease in real time, reflecting subclonal dynamics linked to the heterogeneity of neoplasms or the development of new cancer cell clones and metastases^25^.

However, technical challenges that are mainly related to the small amount of cancer DNA found in cfDNA remain. The use of technologies, such as NGS and ddPCR, partially overcome this problem and allow for the detection of mutations that are present in DNA at fractions as low as 1%. Such technologies have also been used to detect *ESR1* mutations in metastatic BC^15,17,19,26–28^. However, the sensitivity of these technologies is dependent on the quality and quantity of DNA isolated from plasma, making difficult to identify mutations that are present at very low frequencies. Methods aimed at enriching mutant alleles are aimed at overcoming this limitation; to that end, E-ice-COLD-PCR was developed for the enrichment of mutated *KRAS* and *BRAF*^21,23^.

In the present study, we employed NGS or ddPCR methods for the detection of mutant *ESR1* alleles after performing an E-ice-COLD-PCR approach, designed to enrich *ESR1* mutations at codons 536–538. The *ESR1* region was suitable for this type of approach because most of the mutations of interest fall within a small region of 9 nucleotides where the oligonucleotide blocker was designed. E-ice-COLD-PCR was able to enrich mutant *ESR1* alleles up to 100-fold, enabling mutations to be detected even if present at only 0.01% in the initial sample. By using this strategy, we could detect *ESR1* alterations in cfDNA at a sensitivity that could not be achieved by any of the currently employed approaches^4,15–19^, which can reveal the presence of *ESR1* mutations in cfDNA at allelic frequencies generally greater than 5%, with few cases showing mutant allele frequencies at 1–2%, a limit that is mainly related to the high error rates at detection frequencies below 1%. One additional advantage of this approach, compared to using ddPCR or NGS alone, is in the interpretation of the results. The approach makes easier to distinguish positive samples from background noise, even in cases of low-frequency mutations, thus allowing the attainment of clear results also in challenging samples with low frequency alterations or little cfDNA.

By applying the approach to the analysis of clinical samples, we demonstrated the efficacy of our method by detecting *ESR1* mutations in the plasma DNA of patients with metastatic BC. From the analysis of 56 cfDNAs, we found different *ESR1* mutations (L536H, Y537S, D538G, Y537N, Y537H and L536Q) in 15 samples (27% of the total). This confirmed that our method, coupled with NGS, is effective in enriching and detecting all possible alterations present in *ESR1* codons 536–538 without requiring prior knowledge of these alterations.

Notably, *ESR1* mutations were more frequently detected in cfDNA than in biopsies (27% vs. 15%, respectively). Similar results were also obtained in previous studies^18,27^, and the *ESR1* mutation frequency in our investigation was consistent with that reported in a similar study^2^, suggesting that the analysis of tissue biopsies cannot fully represent the heterogeneity of primary tumors or of metastatic lesions; rather, such heterogeneity is more faithfully represented in the ctDNA present in plasma.

In 6 of the patients, it was possible to analyze and compare the mutational status of *ESR1* in both metastatic samples and cfDNA. In other cases, either patient was not alive, precluding the possibility to obtain plasma samples, or only primary tumor biopsy was available. Data from matched biopsies and cfDNAs revealed identical results in 3 patients, but exhibited heterogeneity in the other 3. In the 2 patients (S-28 and S-26) who showed a wildtype *ESR1* according to biopsies but a mutated gene in cfDNAs, the differences were related to the heterogeneity of the tumor sample, or the evolution of the neoplasm over time. Such evolution was clearly shown for patient S-26, where the appearance of the *ESR1* mutation was observed over the 1-year period while the patient was on AIs. Conversely, patient S-51 showed a Y537C mutation in her metastasis biopsy sample, but not in cfDNA that was obtained approximately 3 years later. This patient was treated with fulvestrant during that period, presumably leading to the elimination of the mutant subclone, consistent with the evidence that the Y537C mutation has a modest effect in inducing resistance to fulvestrant and AZD9496^29^. These results illustrate the clinical benefits of cfDNA analysis to monitor *ESR1* gene mutation status in patients with BC. As opposed to single biopsies, cfDNA analysis allows the observation of multiclonal evolution across all lesions.

In conclusion, we report a new approach for a highly sensitive detection of mutations at *ESR1* codons 536–538 in plasma DNA. The method is highly sensitive and specific and can achieve the detection of mutant alleles even when tiny amounts of ctDNA is present in plasma. Here, we have shown that this liquid biopsy approach could be used to monitor patients with metastatic ER+ BC and follow their disease in real time in order to eventually adjust therapies. Given its high sensitivity, this method can also potentially be applied to the monitoring of ER+ non-metastatic BC patients for the early detection of tumor clones that develop resistance to endocrine therapy.

## Materials and Methods

### Patients

Primary tumors and their matched metastases were collected from 40 patients with ER+ metastatic breast cancer who underwent surgical excision of their tumors between 2000 and 2015 at the St. Anna Hospital (Ferrara, Italy). The clinicopathological features of the patients are summarized in Table 1. None of the patients had metastases at diagnosis; however, all patients developed metastasis and recurrence during the course of endocrine therapy. Pathological features were all assessed at the Clinical Pathology Unit of the St. Anna Hospital (Ferrara, Italy) using standard criteria.

Plasma samples were collected from 56 ER+ metastatic breast cancer patients. Among these, 6 were from the first cohort of 40 patients. The study protocol was approved by the *Comitato Etico Unico della Provincia di Ferrara* ethical committee, and written informed consent was obtained from all patients. All participants included in the study were anonymized by using sample identifiers that could not be associated with any individual.

### DNA extraction

Archival formalin-fixed and paraffin-embedded (FFPE) tissue blocks were retrieved, whereupon 10 μm sections were stained with hematoxylin and eosin and were then macrodissected to obtain >70% of the tumor. DNA was extracted from 2 sections of 10 μm using the Maxwell rapid sample concentrator (RSC) instrument (Promega) and Maxwell RSC DNA FFPE kit (Promega), following the manufacturer’s instructions.

We collected 5 mL of blood in EDTA tubes and processed within 4 hours. Plasma was prepared by centrifugation at 1,000 *g* for 10 min and stored at −80 °C. DNA was extracted from 1 mL of plasma using the Maxwell RSC instrument (Promega) and Maxwell RSC ccfDNA plasma kit (Promega), according to the manufacturer’s instructions. DNA was quantified using the Qubit dsDNA HS Assay Kit (Thermo Fisher) on the Qubit 2.0 Fluorimeter (Thermo Fisher Scientific).

### Capillary Sequencing

Sanger sequencing was performed by IGA Technology Services (Udine, Italy) according to standard procedures. Amplicons were generated using the following primers: *ESR1*_192F: GCTCGGGTTGGCTCTAAAGT and *ESR1*_192R: CTTTGGTCCGTCTCCTCCA. Sequences for both the forward and reverse strands were obtained.

### E-ice-COLD-PCR

Primers for E-ice-COLD-PCR were as follows: *ESR1*_109F: AGTCCTTTCTGTGTCTTCCCA and *ESR1*_109R: TCCAGCATCTCCAGCAGC, which amplified a 109 bp PCR product. The oligonucleotides were synthesized by Integrated DNA Technologies (IDT). The 28-nucleotides locked nucleic acid (LNA) blocker had the following sequence (LNA nucleotides are marked with a + ; 3Phos is a phosphate group added to the 3′ end of the molecule): *ESR1*_28_AS_LNA: AGCATCTCCAGCAGCAGG+T+CA+T+AGAGGG/3Phos/. The blocker was synthesized by Exiqon (Denmark). The reverse primer (*ESR1*_109R) and LNA-blocker had an overlap of 15 nucleotides. Theoretical melting temperatures of LNA-blocker/wild-type *ESR1* and LNA-blocker/mutated *ESR1* duplex were determined by using the IDT Biophysics calculator (Integrated DNA Technologies, http://biophysics.idtdna.com/).

E-ice-COLD-PCR was performed in duplicate on 10 ng of genomic DNA from tissues or on 6 μL of cfDNA (from 1 to 10 ng, mean = 2.95 ng, median = 1.8 ng) in a 12.5 μL reaction composed of 1 × Precision Melt Supermix (Bio-rad Laboratories), 100 nM of each primer *ESR1*_109F and *ESR1*_109R, and 80 nM *ESR1*_28_AS_LNA. The reaction was performed on a CFX real-time thermocycler (Bio-Rad), using the following protocol: 2 minutes at 95 °C, 6 cycles of pre-amplification (10 seconds at 95 °C, 30 seconds at 59 °C, and 30 seconds at 72 °C), 49 cycles of the E-ice-COLD-PCR protocol (10 seconds at 95 °C, 30 seconds at 70 °C, 20 seconds at the critical temperature of 80.3 °C, 30 seconds at 59 °C, and 10 seconds at 72 °C), and a final melting curve analysis from 65 °C to 95 °C (5 acquisitions per degree).

### ddPCR

ddPCR was used to evaluate the frequency of Y537S mutations in genomic DNA or E-ice-COLD amplicons. Fluorescent LNA-probes specific for Y537S (56-FAM/CCC+CT+C+T+C+TGAC/3IABkFQ) and wildtype *ESR1* (5HEX/C+CT+C+T+ATG+A+CC/3IABkFQ) sequences were designed and synthesized by IDT (LNA nucleotides are marked with a + ). Droplet digital PCR reactions were set up in a multiplex assay in 20 μL using 1 × ddPCR Supermix as probes (Bio-Rad), 450 nM of each primer *ESR1*_109F and *ESR1*_109R, 100 nM of each probe for Y537S and wildtype *ESR1*, and 20 ng of genomic DNA or 10^–7^-diluted E-ice-COLD-PCR amplicons as templates. Emulsions were created by using a QX200 droplet generator (Bio-Rad), according to the manufacturer’s instructions. Emulsified PCRs were run on a T100 thermal cycler (Bio-Rad) using the following settings: 10 minutes at 95 °C, 40 cycles of amplification (30 seconds at 94 °C, 1 minute at 59 °C, and 10 minutes at 98 °C) setting the temperature ramp increment to 2 °C/second for all steps. Samples were read on a Bio-Rad QX200 droplet reader (Bio-Rad) with QuantaSoft v1.7.4.0917 software (Bio-Rad). The fraction of Y537S mutations was calculated considering the number of Y537S-positive droplets/total number of positive droplets.

### NGS

NGS using an Ion Torrent Personal Genome Machine (PGM) was performed to evaluate the frequency of *ESR1* mutations in FFPE genomic DNA and E-ice-COLD amplicons. Ten nanograms of FFPE genomic DNA were first amplified using Accuprime Taq DNA polymerase (Thermo Fisher Scientific) in a 10 μL reaction using *ESR1*_109F and *ESR1*_109R primers (400 nM final, each). *ESR1* amplicons from each sample were linked to Ion Torrent-specific oligonucleotide motifs to prepare the sample library. Equimolar amounts of each library were pooled and sequencing was performed using an Ion PGM Hi-Q Sequencing Kit (Thermo Fisher Scientific) on an Ion 314 chip, according to the manufacturer’s protocol. Sequencing data analysis was performed as previously described^30^. A sequencing depth of at least 1,500 reads per segment was achieved.

### Data availability statement

Data are all presented in the manuscript. Primary data are available upon request.

## Electronic supplementary material

Supplementary Information
