# Supplementary material for: High-sensitivity assay for monitoring ESR1 mutations in circulating cell-free DNA of breast cancer patients receiving endocrine therapy
Source: Sci Rep. 2018 Mar 12;8:4371. doi: 10.1038/s41598-018-22312-x (PMC5847549; doi:10.1038/s41598-018-22312-x)
Supplement: Supplementary file 1 — Supplementary Information [file 41598_2018_22312_MOESM1_ESM.pdf]

# High-sensitivity assay for monitoring *ESR1* mutations in circulating cell-free DNA of breast cancer patients receiving endocrine therapy

**Laura Lupini, Anna Moretti, Cristian Bassi, Alessio Schirone, Massimo Pedriali, Patrizia Querzoli, Roberta Roncarati, Antonio Frassoldati, Massimo Negrini**

## SUPPLEMENTARY INFORMATION

### Supplementary Figure 1

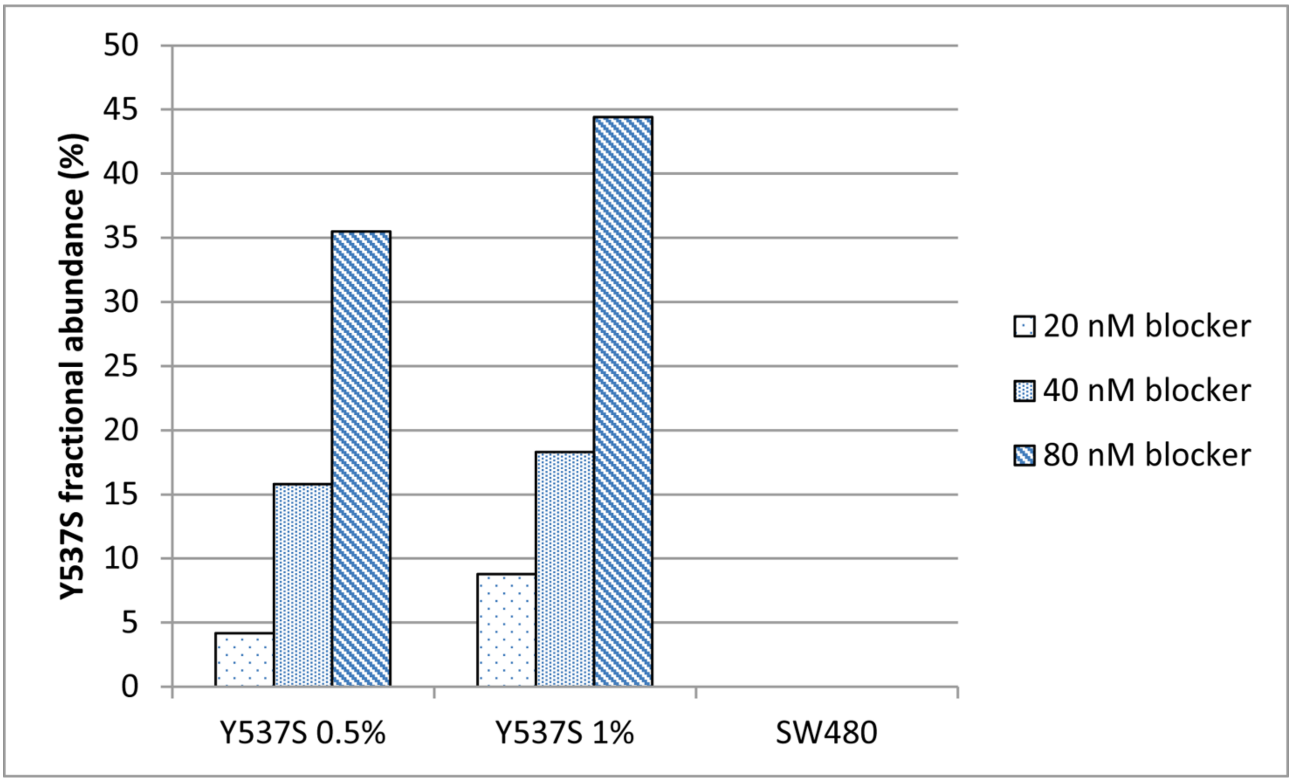


**Supplementary Figure 1. Y537S mutation abundance using different concentration of *ESR1*_28_AS_LNA blocker in E-ice-COLD-PCR assay**. DNA harboring Y537S at the starting frequency of 0.5%, 1% and 0% (SW480) were subjected to three different E-ice-COLD-PCR protocols using respectively 20, 40 and 80 nM of *ESR1*_28_AS_LNA blocker. Amplicons were analyzed through ddPCR, using allele-specific fluorescent probes. A highest enrichment of the mutation was achieved by using 80 nM blocker in E-ice-COLD-PCR.

**Supplementary Figure 2.**

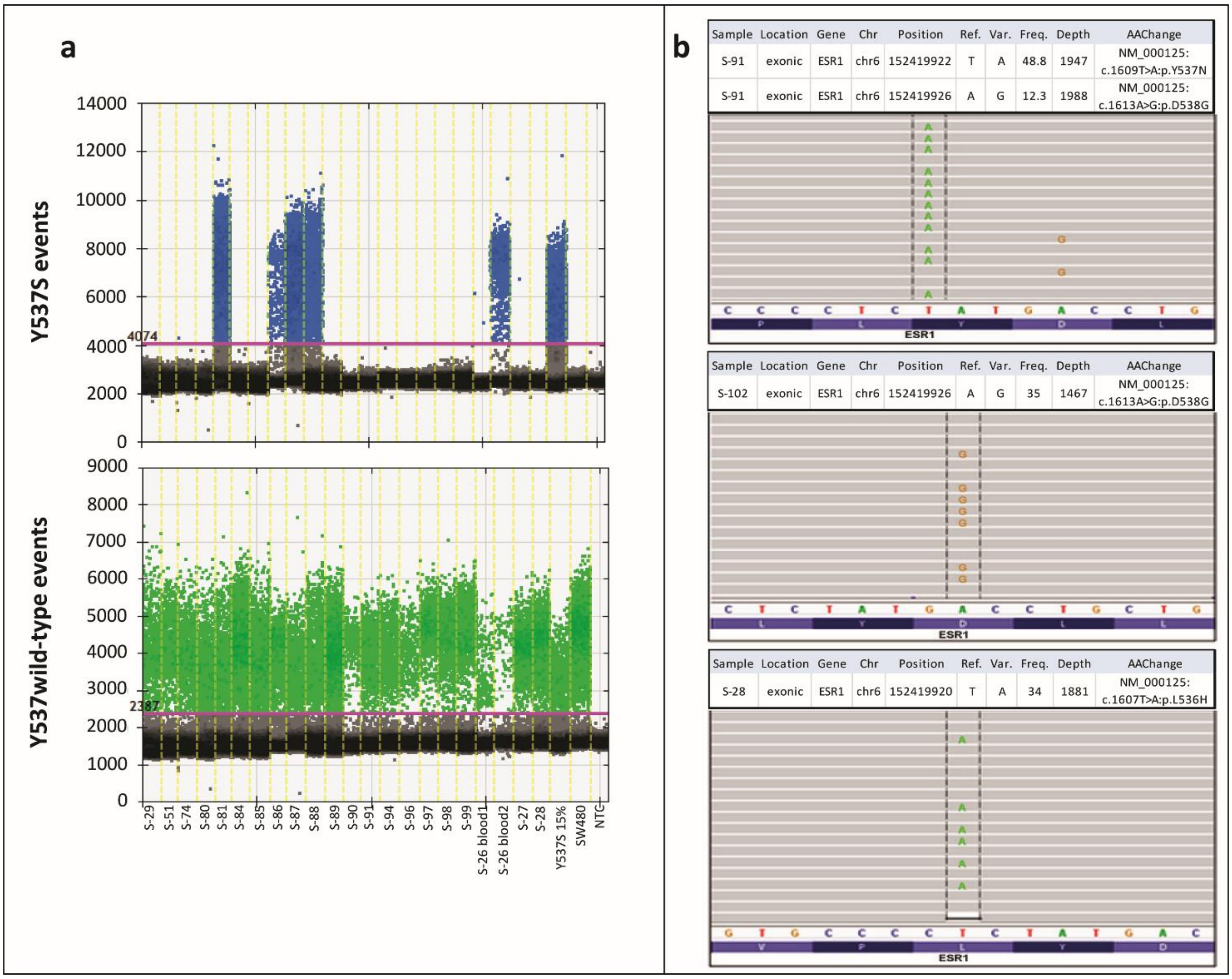


**Supplementary Figure 2. Detection of *ESR1* mutation in cfDNA from mBC patients, following E-ice-COLD-PCR enrichment.** All cfDNA from plasma of mBC patients were subjected to E-ice-COLD-PCR amplifications for the *ESR1* hotspot region and amplicons were analyzed for the presence of mutation by ddPCR (**a**) or NGS (**b**). **a)** The image represents the plots of the *ESR1*-Y537S-positive droplets (blue) and the *ESR1*-wt-positive droplets (green) for a subset of samples. Negative empty droplets are grey. **b)** The image shows NGS results of three samples harboring mutations in the *ESR1* gene. Because of E-ice-COLD-PCR enrichment, samples harboring *ESR1* mutations could be clearly distinguished from negative ones both in ddPCR and in NGS.
